# A Collaborative Approach to the Development of Multi-Disciplinary Teams and Services for Child and Adolescent Mental Health in Uganda

**DOI:** 10.3389/fpsyt.2020.579417

**Published:** 2020-11-03

**Authors:** Godfrey Zari Rukundo, Joyce Nalugya, Patrick Otim, Alyson Hall

**Affiliations:** ^1^Department of Psychiatry, Mbarara University of Science and Technology, Mbarara, Uganda; ^2^Department of Psychiatry, Makerere University, Kampala, Uganda; ^3^Mulago National Referral Hospital, Kampala, Uganda; ^4^Naguru Regional Referral Hospital (China Uganda Friendship Hospital, Naguru), Kampala, Uganda; ^5^East London National Health Service (NHS) Foundation Trust, London, United Kingdom

**Keywords:** child, adolescent, mental health, Uganda (sub Saharan Africa), multi-disciplinary

## Abstract

**Background:** Children and adolescents are especially vulnerable to mental, neurological and substance use disorders during various stages of their growth and development. They often require specialized personnel whose training is time consuming and costly. Consequently many children and adolescents remain untreated in developing countries. This paper describes steps Uganda is taking to develop local capacity for child and adolescent mental health services through training of multi-disciplinary teams.

**Methods:** A 2 year training programme was introduced in accordance with the Ugandan Ministry of Health Child and Adolescent Mental Health Strategy. This had been jointly developed in 2008 by Mbarara University of Science and Technology, Makerere University, the Uganda Ministry of Health and East London Foundation NHS Trust, United Kingdom (UK). The initial funding for the programme focused on monitoring and evaluation of the training, quality of clinical practice and clinical activity data.

**Results:** Fifty health workers have been trained and are now working at regional referral hospitals and non-governmental organizations. Monitoring and evaluation demonstrated major increases in the range of disorders and client numbers (2,184–31,034) over 6 years. There was increased confidence, knowledge and skills in assessment. Learning in a multidisciplinary environment was interesting and helpful. Assessments were more thorough and child centred and more psychological treatments were being used. Programme graduates are now contributing as trainers.

**Conclusion:** The clinically focused multidisciplinary training has yielded rewarding outcomes across Uganda. Ongoing support and collaborative work can expand service capacity in child and adolescent mental health for Uganda and other developing countries.

## Introduction

Children and adolescents are vulnerable to certain specific mental, neurological and substance use disorders (MNS) due to their incomplete physical, mental and social growth and development (1–3). The most common MNS disorders include developmental disorders such as intellectual disability, autism, attention deficit hyperactivity disorder, elimination disorders; neurological disorders such as epilepsy; emotional disorders such as anxiety, depression, post-traumatic stress disorder, conversion disorders, specific phobia, school refusal and behavior disorders such as oppositional defiant disorder. Conduct disorder, substance use disorders and psychosis are more common in adolescence (4, 5). Overall boys are generally more affected than girls (6–9). In general, girls tend to have more emotional disorders and psychosis especially in adolescence while developmental, behavioral and conduct disorders are more common in boys. The affected children are usually not well integrated into educational, community and social programmes (10). As a result, affected individuals are less likely to develop their full potential in order to live productive adult lives (10). Child and adolescent mental health problems present in broadly similar ways globally but there are specific differences associated with LMICs. For example epilepsy is two to five times more prevalent in sub-Saharan Africa where malaria is prevalent and in many of these countries it may be managed as a mental health disorder rather than by pediatricians (11).

Childhood MNS disorders have unique presentations and their treatment differs significantly from those disorders first presenting in older adolescents and adults (12–14). These varied presentations during childhood are due to brain development, expressions and risk factors at different stages of development and include the consequences of abuse and neglect and separation from parents or caregivers. Assessment of the child's development, family relationships, parenting and educational issues is essential. Children need a more flexible approach to assessment because they may not be able to explain their symptoms, feelings and troubling events. They may require more time and techniques like drawings, storytelling, drama or play. Specialist personnel are required for proper assessment and management (15) and to provide training in the recognition of disorders for primary care professionals and teachers. Training such personnel requires resources and expertise not generally available in LMICs so that most children and adolescents with mental health problems, especially in countries like Uganda, remain unrecognized and untreated (16–18). Uganda, like other developing countries has very few child and adolescent psychiatrists serving a population of over 25 million children and adolescents (16, 19, 20). Four child and adolescent psychiatrists were trained in UK, Belgium and South Africa while two have so far been trained in Uganda. Following a recent retirement, the remaining five child and adolescent psychiatrists all carry significant additional responsibilities including university teaching, administration and adult psychiatric services. Clearly, the development of CAMH services for such a large population would require additional trained professionals to provide the backbone of assessment and treatment throughout the country (21).

In Uganda many children suffer psychological trauma from war, abuse and bereavement but in common with other LMICs (16), few children with emotional and behavioral disorders reach services. Developmental problems such as attention deficit disorder or autism have rarely been recognized. Epilepsy is common, often causing brain damage, accidental injury and rejection and is overrepresented in clinic attendances. At the only dedicated CAMH clinic in 2008 over 90% of children presented with epilepsy and a few with intellectual disability. Admissions to the adult wards of the hospital were almost all older adolescents with psychosis or younger children with intellectual disability of unknown identity found abandoned by the police (22). Apart from adolescents with psychosis, those whose problems were recognized are not treated by specifically trained professionals. Some are treated by traditional healers but the majority are managed within mental health services in regional hospitals and community health centres by Ugandan mental health nurses and psychiatric clinical officers, a grade of prescribing professionals mostly from a nursing background. They had received minimal training in CAMH or child health and, when treating children, generally relied on psychotropic medication used for adult disorders. This was appropriate for older adolescents with psychosis or affective disorders but the use of medication, for example haloperidol or diazepam for autism, intellectual disability or attention deficit disorder in young children could be harmful to their development, health and education. Psychological treatments such as family and behavior therapy were unavailable.

Stigma is one of the challenges facing children and adolescents with mental disorders (23), especially those with epilepsy or intellectual disability who may be abandoned by their families. One of the ways to address stigma is to integrate child mental health services into primary health care (24). This would be followed by training health professionals at primary care level to assess and treat childhood mental health problems. This can only be possible once there is sufficient capacity of trained CAMH professionals to do this (25).

This paper describes steps Uganda is taking to develop local capacity for child and adolescent mental health services through training of multi-disciplinary teams with the introduction of a university accredited Advanced Diploma in CAMH for public health workers and national CAMH policy guidelines. The process, achievements and results of UK project monitoring and evaluation are described with discussion of challenges faced and relevance to other LMICs.

## Methods

### Background

In 2008 a national strategy for child and adolescent mental health in Uganda was developed by a partnership of the Faculties of Medicine of Mbarara University of Science and Technology, Makerere University, Ugandan Ministry of Health and the Butabika East London Link (a link between Butabika National Psychiatric Referral Hospital and East London Foundation NHS Trust, UK). The aims of the strategy were to develop a specialist child and adolescent mental health service for Uganda by providing dedicated out-patient and in-patient care with trained specialists at regional level and developing a multi-disciplinary workforce that can train professionals in health and education at district level to recognize child mental health problems and educate the population in prevention. The core requirement of the strategy was specialist training. In order to introduce a national training programme four Ugandan professionals were trained in the UK on Commonwealth Scholarships when a draft curriculum was jointly drafted.

### UK Funded Projects

The Butabika Link East London Link obtained funding for two projects (2012–2017) from the Health Partnership Scheme of the Department for International Development, UK government, to develop and implement a multi-disciplinary training programme in CAMH for mental health and child health professionals in Uganda. The majority of the training in the initial course was provided by UK CAMH professionals but the second project evaluated the delivery of the course by the new cohort of trained Ugandan professionals and secured university accreditation from Mbarara University of Science and Technology as the Advanced Diploma in CAMH. The initial training programme was preceded by a training the trainers course and further training of trainers took place in preparation for the Ugandan led course. Exercises, focus groups and discussion allowed for anxieties about the heterogeneous nature of the multidisciplinary participants to be considered and managed during the training programme.

The Butabika Link East London Link obtained additional UK government funding for two smaller projects (2016–18) to assist the Ugandan Ministry of Health and CAMH professionals to develop, launch and implement a national CAMH Policy.

### Programme Description

The diploma is a 2 year modular programme with the six first year courses providing basic training that focus on practical skills in child and family assessments, and recognition and management of mental disorders in children and adolescents. The first year of the training serves as a foundation for the second year courses which focus on psychological therapies, clinical supervision, training, service development and research. Each module consists of 5 days of classroom teaching at the Butabika School for Psychiatric Officers followed by 5 days of clinical experience in the specialist children's ward at Butabika Hospital. The trainees are expected to focus on assessing and treating children and adolescents in their workplace between modules and to complete case studies, a logbook and a research or audit project. As well as written examinations for each course, clinical and teaching skills are examined. The programme provides training that equips trainees with the knowledge, clinical, teaching and managerial skills to provide care, develop child mental health services and to train others. The criteria for admission to the programme include commitment, professional mix and regional representation, and ability to teach others. Previous training/expertise and interest in child and adolescent mental health are key.

### Monitoring and Evaluation (M&E)

Apart from assessment of the trainees' academic and clinical performance, M&E for the training projects consisted of evaluation of the quality of the course by trainees (individual structured feedback forms and focus groups) together with evaluation of the impact of the training on service delivery and quality of clinical work over 6 years. Baseline activity data was collected at the four teaching hospitals at the start of the first project and as trainees joined the course from other locations an M&E coordinator collected the number of child attendances per month from a professional at each site by age group for out-patient attendances and admissions. This included a record of children seen by both CAMH and non CAMH staff. The number of dedicated clinics for children and of CAMH trained staff at each location was recorded.

The quality of clinical work was assessed on an individual basis during clinical training and examined twice. A sample of the twenty most recent case notes for outpatient attendances or admissions were assessed annually at each of four main sites using a case note monitoring form. This recorded patient demographics, diagnosis, assessing professional (CAMH trained or not), indicators of quality of assessment (child development, education, medical and family information, genogram, observation of child and relationships), management and appropriate use of medication (no prescription when not indicated). For follow ups it recorded use of psychological treatments, reviews of diagnosis and management including medication side effects.

## Results

### Professionals Trained in CAMH

The first two diploma cohorts funded by the projects completed their training in 2015 and 2017. Since then the first and second year courses have been running simultaneously allowing a new cohort to graduate each year with more efficient use of teaching resources. Fifty health workers from various medical backgrounds have been trained since 2012 and are working with children at various regional referral hospitals. These comprise two psychiatrists, one medical officer, ten psychologists, sixteen psychiatric clinical officers, twelve nurses, two occupational therapists, five social workers/counselors, one public health officer and one human resource officer (males 64% and females 36%). These new cohorts of trained CAMH professionals have been able to contribute to the training programme, provide peer – peer mentoring and supervision as well as providing clinical assessments and management of children with mental health and developmental disorders at their work places (Table 1).

**Table 1:** Types and regional location of CAMH trained health workers.

**Cadre**	**Gender**		**Total**	**Region**				
	**M**	**F**		**Central**	**East**	**West**	**North**	**South**
Psychiatrist		2	2	2				
Medical officers	1		1		1			
Psychologist	6	4	10	6		1	3	
Psychiatric clinical officers	13	3	16	8	4	2	2	
Occupational therapy	2		2	2				
Psychiatric nurse	4	4	8	4		1	3	
General Nurse	3	1	4			2	1	1
Public health officer	1		1				1	
Social worker	1	4	5	1	1	3		
Human resource officer	1		1	1				
TOTAL (%)	32 (64)	18 (36)	50	24	6	9	10	1

### Quality and Experience of Training

Monitoring and evaluation of the training programme showed that following the training, the trainees developed increased confidence, knowledge and skills in assessment and management of mental health problems in children and adolescents and in teaching and presentation skills. All the trainees study together but different professional groups have varied levels of experience related to topics in the programme and share their knowledge and experience. The roles of specific professions are discussed in depth at the end of the programme (for example psychometric assessment for psychologists). Initially the professionals found learning in a multi-disciplinary group unfamiliar and some were uneasy about different educational levels, for example between doctors and nurses. The training groups quickly became cohesive and mutually supportive which was crucial for trainees working in isolation around the country. Individuals spontaneously commented how valuable it was to train with and learn from other professionals from social work, psychology, occupational therapy, counseling, and child health. This enriched trainees' knowledge and skills through shared experiences and they came to see the importance of developing a multidisciplinary and inter-professional environment in child mental health services.

Some quotes from trainees following clinical placements included

“*I am building more relationship with my clients and they keep the appointments. I have noticed that the way we ask questions is indeed very important and that when you begin the interaction with your clients with a smile and have a smile during and at the end of the session it becomes more therapeutic. Please members let us try it!!!” (CAMH Trainee)*

“*I did receive a child aged 13 yrs with history of shaking the right arm for about a month. I applied the skills that I had learnt including questioning skills, history taking, and genogram. I was amazed when the mother commented that she had never met a doctor who could ask her such questions. She was impressed and interestingly the girl left my clinical room when the shaking had stopped. The mother telephoned me yesterday thanking me and saying that the girl has been free from shaking for the last five days.” (CAMH Trainee)*

“*I would like to thank you for all the efforts, work and time that you have dedicated to our training. Surely there is no enough thank you or reward that I can give, except results of the practice of the skills that I have learnt over this period of time. At my new workplace, all my colleagues know that I have speciality in CAMHS and have been booking patients for me which is a great start in a new place.” (CAMH Trainee, now trainer)*

“*The course in child and adolescent mental health provided a deserving opportunity for the first trainees in Uganda to acquire knowledge and skills in this field so as to provide a service that was previously non-existent in most hospitals in this country. The course content effectively addressed the common mental health problems in children and adolescents in our community. In future trainings more emphasis should be placed on psychotherapies, family therapy and child protection. There is however, a huge need for child and adolescent mental health service in this country and therefore the need to train more child and adolescent mental health specialists.” (CAMH Trainee, now trainer)*.

### Service Development

The final project was completed in 2019 and monitoring and evaluation for the projects demonstrated that, since the start of the Diploma course in 2012, the number of sites with a trained CAMH professional increased from 4 to 24 with all regions of the country represented. The number of children seen in their services increased from 2,184 to 31,034 (Table 2).

**Table 2:** Changes in CAMH activity and service capacity since 2012.

	**2012**	**2017**	**2019**
Trained CAMH professionals	5	36	50
Children attending services with	2,184	25,480	31,034
a CAMH professional	(at 4 sites)	(at 14 sites)	(at 24 sites)
Specialist CAMH clinics	3	36	37

The development of multidisciplinary teams has so far been confined to the university hospitals while CAMH trained mental health and child health professionals provide much of the service to children in regional hospitals and health centres around the country as well as continuing to see adults. Despite there being just one or two CAMH staff at these units, most were able to set up specialist CAMH clinics by dedicating specific clinic days for children. The specialist multidisciplinary teams at the national referral hospitals in Kampala, Butabika, and Mulago, have been able to provide daily specialist CAMH outpatient services and a large children's inpatient service at Butabika, A few CAMH Diploma graduates have been employed by NGOs to work with refugee children mainly in Northern Uganda where their skills have also been utilized extensively in staff training.

### Service Quality

In addition to the substantial increase in children attending services, examination of clinical records showed more children were having thorough child centred assessments, and there were increased numbers and range of non-epilepsy diagnoses compared to baseline findings. The improved quality of assessments by CAMH trained staff compared with assessments by other mental health professionals included the recognition of co morbidity in epilepsy cases, less medication used, more appropriate medication used, increased use of psychological treatments and more revisions of diagnosis and management for cases seen by non CAMH professionals. For example, assessments now included observation of the child, child's views, their drawings, and observations of family relationships accompanied by a drawing of the family genogram. Less medication was being prescribed whereas before the training, most children with mental health problems were given psychotropic medication due to lack of adequate knowledge of psychological treatments. All pioneer graduates of the programme have been equipped with training skills through training the trainers courses and have been providing training for large numbers of health and primary care workers, teachers and community groups in their local areas.

Peer support supervision has been piloted over 2 years around the country and has worked well to support CAMH workers in the regions and provide continued professional development for local health professionals. There has been great enthusiasm for continuing the 2 day events but sustaining them without funding for transport is a problem. Butabika Hospital already has a professional supervision system for PCOs working in the regional hospitals and funding has been sought from the Ministry of Health for a separate specialist supervision system for CAMH workers.

### National Perspective

In 2017 the Ministry of Health launched the Ugandan Policy for Child and Adolescent Mental Health (26). Other achievements include two National CAMH conferences in 2015 and 2017 with 135 and 165 delegates, respectively, mainly from Uganda but also from other countries. Ten scholarships were provided though the Ministry of Health for new trainees for 2017–2019 and six scholarships for 2018–2020.

“*This training is timely, not just being academic but responding to one of Uganda's biggest public health problems. It is a speciality that needs to be recognised and embraced at all levels” (Official, Ugandan Ministry of Health)*.

“*Research interest in the subspecialty has been generated and the development and launch of CAMH policy was a great achievement...... I hope that people will come from neighbouring countries as this training programme is the first of its kind in the region” (Hospital Executive Director)*.

### Independent Evaluation

An independent value for money assessment of the first training project was commissioned by the Tropical Health Education Trust (27). It concluded that the project costs were kept low (£160,000) because of the use of UK volunteers at the trainer, coordinator and management level of the project. The design was appropriate for the needs identified. As well as the successful training of 23 health workers, quality of CAMH services were found to have improved on a number of indicators, including reduced use of medication and introduction of child-dedicated services. High-level official buy-in was demonstrated through the adoption of CAMH protocols, policies and curricula at universities, hospitals and the Ugandan Ministry of Health, including a draft Ugandan CAMH policy. Sustainability was ensured through funding for the transition of the training programme to an accredited University Diploma, to be taught by the Diploma graduates. Equity was demonstrated at the participant and beneficiary level, from the choice of rural and urban hospitals spread across Uganda, to the gender and country balance found at the senior decision-making level.

## Discussion

Development of the CAMH training programme in Uganda and a multidisciplinary service for most regions of Uganda has had a major impact on the numbers of specialist CAMH staff, attendances by children with mental health problems and the quality of treatment received but has not been without challenges. The extensive preparatory work, need for external funding, achievement of university accreditation and current challenges in relation to workload and sustainability are relevant to the development of specialist clinical training programmes in other LMICs (18, 22, 25). The support received from the outset from the Ugandan Ministry of Health and the health partnership link with the UK have been invaluable.

Following the strategic work that took place with the Ministry of Health in 2008, before the training programme could start in 2012, it was necessary to train four key staff in the UK on Commonwealth Fellowships, develop a curriculum to meet university regulations and secure funding for the first UK funded training project. The first two fully funded groups graduated in 2015 and 2017 but university accreditation for the Advanced Diploma in CAMH was not achieved until 2017.

The Ministry of Health has recognized the diploma as the specialist qualification for child mental health posts in Uganda and this has been supported by the introduction of the MOH Policy for CAMH (26) and its implementation initiated in 2018. Despite this the number of specialist CAMH posts has not yet increased which affects service delivery for children outside the two national referral hospitals, Mulago and Butabika.

### Service Challenges

Prior to the training programme very few children attended mental health services and those who did mainly suffered from epilepsy and intellectual disability. Training of local hospital and community health staff by CAMH professionals and public education through the media and community groups has resulted in increased recognition and referral of a range of mental health problems. This presented anticipated challenges (25, 28), with an overwhelming increase in numbers of children and adolescents attending regional mental health units and health centres where most CAMH trained staff are still responsible for the care of adults. The specialist clinics for children remain based in adult mental health units and children are admitted to adult wards except at Butabika. Admissions have increased substantially throughout the country because some children travel long distances and require admission to initiate treatment. CAMH professionals have also made a significant contribution to the mental health of child refugees both within the public health system and working with NGOs in the camps where their specialist clinical and teaching skills have been valued.

In such overstretched services with few staff almost half of child cases were still seen by non CAMH staff although we found that younger children and new cases were more likely to be seen by CAMH staff. It has been important to prioritize new cases and young children for specialist CAMH assessment. Adult trained staff are familiar with the management of older adolescents with psychosis, affective disorders or substance misuse.

### Recruitment

Because of the large and growing child population in Uganda including many refugees with high levels of need, it is important to train more child and adolescent psychiatrists, accessible in rural areas, to support CAMH professionals working in isolation as well as pediatricians and child health workers who see many children with mental health problems. They can be recruited from both psychiatry and child health.

General adult psychiatrists are eligible to undertake the training to become child and adolescent psychiatrists and we continue to lobby the MOH to support posting of medical officer special grade (psychiatry) to the regional and district hospitals. Those interested in providing CAMH services may then access the MOH scholarships for the Advanced Diploma in CAMH. Since the training programme was opened to child health professionals a medical officer and a clinical officer in child health have graduated. Pediatricians and medical officers in Uganda and sub Saharan Africa have shown interest in the training and some of these may then undertake psychiatric training in order to be eligible for consultant posts. To this end it will be important to publicize the training to attract international applicants from countries with few trained CAMH staff.

In addition to trained doctors, a sustained increase in the capacity of the multidisciplinary CAMH workforce will be essential because of the growing volume of work and the unmet need for access to specialist CAMH services in all regions of Uganda. Priority for training and scholarships will need to be given to individuals from different professional backgrounds and from poorly served parts of Uganda. The provision of scholarships and the development of specialist CAMH posts will be essential for public health workers who so far have shown tremendous commitment to the specialty and have been very active in training their colleagues and community workers.

### Sustainability

The continued implementation of the national CAMH policy is crucial and, as part of this, CAMH professionals have provided training and held meetings with local directors of regional hospitals. Mental health services in the regions have many unfilled posts and the local promotion of child mental health awareness is vital to convince hospital directors of the need to appoint CAMH staff and to designate vacant posts for CAMH.

Financial sustainability of the course has been a major challenge since the two UK funded training projects ended because public health workers for whom the course was designed, have low salaries and cannot afford the modest university fees, internet and travel costs. Provision of free residential accommodation at Butabika Hospital during the teaching modules has been invaluable for trainees outside Kampala. There were delays in securing scholarships for university fees through the Ministry of Health and in the transference of funds to the teaching institution affecting payment of course expenses and teaching fees. CAMH trainers experienced financial stress while balancing their teaching contribution with demanding service responsibilities. Long term reliable sponsorship for university fees is required otherwise the number of public health workers who can be trained will be limited.

### Implications for Other Developing Countries

Like Uganda many LMICs have a young population with high percentages of children in need of specialist child and adolescent mental health services (15–17, 19). There are limited resources in terms of human capital, finances and infrastructure and often other challenges (19, 25, 28, 29). There is potential to scale up our programme to support the task sharing or task shifting strategy of integrating child and adolescent mental health services into the existing structures in their respective countries. Countries with no child and adolescent mental health specialists could support a few of their health professionals to come to develop specialist clinical skills and train as trainers with the aim of conducting similar capacity building back home. The course is not dependent on the training of medical staff unlike most international CAMH programmes (30) and the modular design allows professionals to remain in their work places for 40 weeks per year, reducing the pressure on hard pressed services and allowing them to put their new skills into practice.

For LMICs planning to develop child mental health services through their own training programme we can make some recommendations drawn from our experiences over 12 years. It would be important to train a few key individuals in CAMH in advance to take both a training and strategic lead in order to develop a countrywide initiative, with support from and involvement of the ministry of health at an early stage. A teaching site ideally in proximity to a clinical training base where large numbers of children can be seen needs to be identified. Rather than a pure academic focus, the training programme needs to aim to develop a high level of clinical skills with associated knowledge and understanding for professionals working in public health services. Ideally it should be linked to a university as soon as possible as obtaining academic approval for such a specialist course was in our experience a long drawn out process. While the programme's demands meant that it could have been offered as a postgraduate master's degree, it was important to be able to recruit a multidisciplinary group including nurses from services throughout the country hence its designation as an advanced diploma. The modular design works well but thought needs to be given to the provision of accommodation and food with the possibility of scholarships to enable low paid health workers to attend. At the outset some seed funding and sponsorship would be likely to be required. We were fortunate to have UK government funding with UK volunteers to develop and evaluate the programme and associated government policy but we hope that other countries will be able to make use of our resources and experience to develop child mental health services themselves (31).

The ongoing partnership with East London provided continued opportunities for long term volunteers from UK and other -high income countries to come to Uganda to support the CAMH programme contribute to training the trainers, deepen skills in psychological therapies and to further develop Butabika as a centre for clinical training. They have benefited from the opportunity to develop new skills in cross cultural working and project management and also to conduct research programmes. The relationships established have proved mutually beneficial with continued electronic and telephone communication to support the training programme.

## Conclusions

Initial steps to train a cohort of CAMH professionals in Uganda have yielded rewarding outcomes. With collaborative effort over time, it proved possible to develop multidisciplinary teams for both urban centres and rural areas through the national and regional referral hospital system. These multi-disciplinary professionals have been able to improve both access to and the quality of mental health services for children and to train others in their local hospital and community services even when working in isolation. The multidisciplinary nature of the programme implies that different categories of staff can be trained; and recently this has included some professionals providing general health care in rural health centres where they can easily be accessed by the rural communities. This means that communities can be trained to identify children with mental health problems as early as possible and refer to appropriate services within their communities or regional centres.

## Data Availability Statement

The data analyzed in this study is subject to the following licenses/restrictions: The raw data from which the article has been written will be available on request to the authors. Requests to access these datasets should be directed to Godfrey Zari Rukundo, grukundo@must.ac.ug.

## Ethics Statement

Ethical review and approval was not required for the study on human participants in accordance with the local legislation and institutional requirements. Written informed consent from the participants' legal guardian/next of kin was not required to participate in this study in accordance with the national legislation and the institutional requirements.

## Author Contributions

GR wrote the first draft of the article. The rest of the authors JN, PO, and AH contributed significantly to the writing of the final paper, improving on the draft. All authors contributed to the article and approved the submitted version.

## Conflict of Interest

The authors declare that the research was conducted in the absence of any commercial or financial relationships that could be construed as a potential conflict of interest.
